# Lithobius (Ezembius) hualongensis sp. nov. and Lithobius (Ezembius) sui sp. nov. (Lithobiomorpha, Lithobiidae), two new species of centipede from northwest China

**DOI:** 10.3897/zookeys.892.39033

**Published:** 2019-11-27

**Authors:** Penghai Qiao, Wen Qin, Huiqin Ma, Gonghua Lin, Tongzuo Zhang

**Affiliations:** 1 Institute of Laboratory Animal Science, Guizhou University of Traditional Chinese Medicine, Guiyang, 550000, China Guizhou University of Traditional Chinese Medicine Guiyang China; 2 Qinghai Provincial Key Laboratory of Animal Ecological Genomics, Northwest Institute of Plateau Biology, Chinese Academy of Sciences, Xining 810008. No.23 Xinning Road, Chengxi District, Xining, Qinghai, China Northwest Institute of Plateau Biology, Chinese Academy of Sciences Xining China; 3 Scientiﬁc Research Oﬃce, Hengshui University, Hengshui, 353000, China Hengshui University Hengshui China; 4 School of Life Sciences, Jinggangshan University, Jian, 343009, Jiangxi, China Jinggangshan University Jian China

**Keywords:** myriapod, Qinghai-Tibet Plateau, stone centipede, taxonomy

## Abstract

Lithobius (Ezembius) hualongensis**sp. nov.** and Lithobius (Ezembius) sui**sp. nov.** (Lithobiomorpha, Lithobiidae) recently discovered from the Qinghai-Tibet Plateau, China are described. Morphologically, the two new species are very similar but can be distinguished by the number of coxosternal teeth: L. (E.) hualongensis**sp. nov.** has 2 + 2 while L. (E.) sui**sp. nov.** has 3 + 3. The two new species resemble L. (E.) multispinipesPei et al., 2016, from the Xinjiang Autonomous Region, but can be readily distinguished by having the Tömösváry’s organ slightly larger than the adjoining ocelli rather than smaller, 3 + 3 spurs on female gonopods versus 2 + 2, and the simple terminal claw of female gonopods with a small triangular protuberance on the basal ventral side versus simple, without a small triangular protuberance on the basal ventral side. We also compare the main morphological characters of the two new species with the other Lithobius (Ezembius) species known in Qinghai Province. A key to the Chinese species of *Ezembius* is presented.

## Introduction

The myriapod fauna of China is still poorly known and this is especially the case with centipedes of the order Lithobiomorpha. Only about 84 species/subspecies of lithobiomorphs are known from the country (Ma et al. 2014a, b, 2015, 2018; Pei et al. 2014, 2015, 2016, 2018; Qin et al. 2014; Qiao et al. 2018a, b, 2019a, b). Qinghai province is among the very poorly studied regions of China with only 11 species at present registered from its territory (Ma et al. 2014b, Qiao et al. 2018a, b, 2019a, b). Altogether, 26 species of Lithobius (Ezembius) have been recorded from China (Zhang 1996; Pei et al. 2018, Qiao et al. 2018b, 2019a, b). Herein we describe Lithobius (Ezembius) hualongensis sp. nov. found in Hualong County, Qinghai and Lithobius (Ezembius) sui sp. nov. collected from Minghe County, Qinghai.

The centipede subgenus Ezembius was erected by Chamberlin (1919) as a genus to receive *Lithobius stejnegeri* Bollman, 1893, *L. ostiacorum* Stuxberg, 1876, *L. princeps* Stuxberg, 1876, *L. sulcipes* Stuxberg, 1876 and *L. scrobiculatus* Stuxberg, 1876 and then was formally proposed as new and described in 1923 (Chamberlin 1923). It accommodates a group of 58 species/subspecies known mostly from Asia, but also western North America and spans a wide range of habitats, from the arctic and sub-arctic to tropical and sub-tropical forests, to steppe and overgrazed stony areas of central Asia, to Himalayan montane forests, from the sea shore up to 5500 m (Himalayas) (Zapparoli and Edgecombe 2011). Most of species are not widely distributed (Bonato et al. 2016), except Lithobius (Ezembius) giganteus Sseliwanoff, 1881 distributed in Mongolia, eastern Kirgizia Buryat and Soviet Central Asia (Eason 1986) and Lithobius (Ezembius) sibiricus Gerstfeldt, 1858 distributed in Asian Russia in Western, Central and Eastern Siberia, the Russian Far East and northern Mongolia (Eason 1976).

*Ezembius* is characterized by antennae with ca 20 articles; ocelli 1 + 4 to 1 + 20; forcipular coxosternal teeth usually 2 + 2, sometimes 2 + 3, 3 + 2 or 3 + 3; porodonts generally setiform but sometimes stout; tergites generally without posterior triangular projections, occasionally with; tarsal articulation of legs 1–13 distinct; female gonopods with uni-, bi- or tridentate claw, 2 + 2 or 3 + 3, rarely 4 + 4, spurs (Zapparoli and Edgecombe 2011). The distinction between *Ezembius* and *Monotarsobius*, depends on the size and state of the anterior tarsal articulations (Eason 1992): *Monotarsobius* has smaller body size never more than 11 mm long and fused tarsal articulation (Zapparoli and Edgecombe 2011).

## Materials and methods

All specimens were hand-collected under leaf litter or stones. The material was examined with the aid of a Motic-C microscope of which the measuring accuracy is +/- 0.01 mm. The color description is based on specimens in 75% ethanol, and body length is measured from the anterior margin of the cephalic plate to the posterior end of the postpedal tergite. Type specimens are preserved in 75% ethanol and deposited in Northwest Institute of Plateau Biology, Chinese Academy of Sciences. The terminology of the external anatomy follows Bonato et al. (2010). The following abbreviations are used in the text and the tables: **a** anterior; **C** coxa; **D** dorsal; **F** femur; **m** median; **p** posterior; **P** prefemur; **S**, **SS** nsternite, sternites; **T**, **TT** n tergite, tergites; **Ti** tibia; **To** Tömösváry’s organ; **Tr** trochanter; **V** nventral; **HL** Hualong County; **MHA** Minhe County.

## Taxonomic accounts

### Order Lithobiomorpha Pocock, 1895

#### Family Lithobiidae Newport, 1844

**Subfamily Lithobiinae Newport, 1844**

**Genus *Lithobius* Leach, 1814**

**Subgenus Ezembius Chamberlin, 1919**

##### Lithobius (Ezembius) hualongensis sp. nov.

Taxon classificationAnimaliaLithobiomorphaLithobiidae

FE5341D6-9D01-5FFB-A458-9C17222550EC

http://zoobank.org/B145889E-4CEC-42E6-8C14-579C9B0616A0

**Figure 1.**
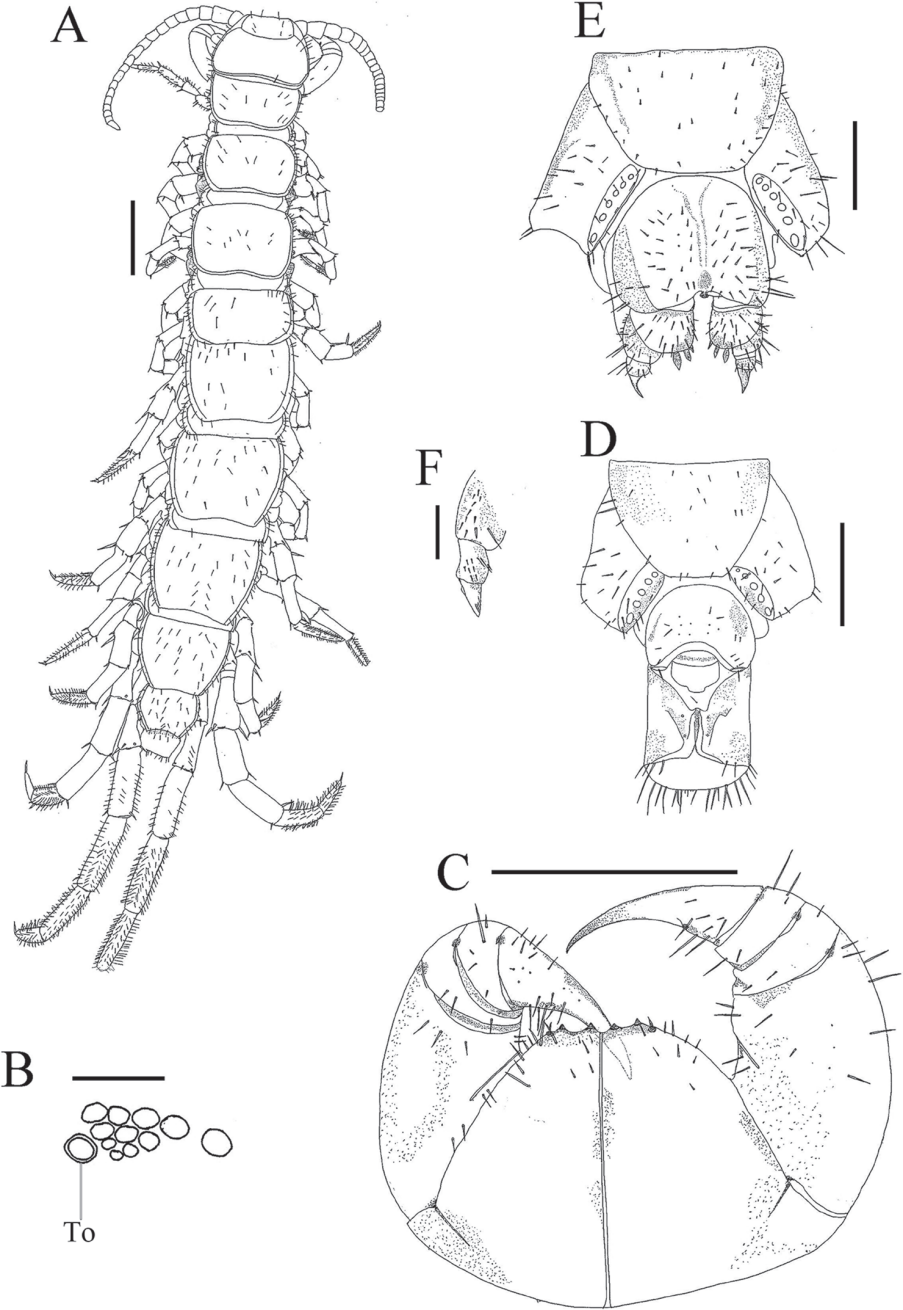
Lithobius (Ezembius) hualongensis sp. nov. **A, C, D** holotype, male: **A** habitus, dorsal view **C** forcipular coxosternite, ventral view **D** male posterior segments and gonopods, ventral view **B, E, F** paratype, female: **B** ocelli and Tömösváry’s organ (**To**), lateral view **E** female gonopods, dorsal lateral view **F** female posterior segments and gonopods, ventral view. Scale bars: 1 mm (**A, C**); 500 μm (**D, F**); 250 μm (**B, E**).

Fig. 1A–F ; Tables 1 , 2

###### Type materials.

***Holotype***: ♂ (HL9), Hualong Hui Autonomous County, Qinghai Province, 36.18848333N, 102.2971333E, 3185 m, a.s.l., 14 April 2012, leg. Lin Gong-Hua, Li Wei-Ping. ***Paratypes***: 1 ♀ (HL7), 2 ♂♂ (HL1, HL4), all from the same locality.

###### Diagnosis.

A Lithobius (Ezembius) species with body length 12.31–16.15 mm, antennae of 20 + 20 articles; 8–11 ocelli on each side, arranged in 3 irregular rows, terminal two ocelli comparatively large; Tömösváry’s organ distinctly larger than the adjoining ocelli; 2 + 2 coxosternal teeth; porodonts posterolateral and ventral to the lateral-most tooth; posterior angles of all tergites without triangular projections; 4–7 coxal pores oval to round, arranged in one row; female gonopods with 3 + 3 moderately large, coniform spurs; terminal claw of the third article simple, with a very small triangular protuberance on basal ventral side; male gonopods short and small, with one long setae on the terminal segment.

###### Description.

**Male** (Fig. 1A). Body length 12.31 mm, cephalic plate 1.15 mm long, 0.92 mm broad.

***Coloration*.** Body and antennae reddish brown. Pleural region pale grey. Sternites yellow-brown. Distal part of forcipules red-brown, with basal and proximal parts of forcipules and forcipular coxosternite yellow-brown. Legs 1–15 pale yellow-brown.

***Antennae*** composed of 20 + 20 articles extending back to anterior part of T3, basal article about the same width as length, second article longer than wide, third article slightly longer than wide, with following articles tapering, distal-most article 2.7 times as long as wide; abundant setae on antennal surface, gradual increase in density of setae to about 4^th^ article, then more or less constant.

***Cephalic plate*** smooth, cordiform. Frontal marginal ridge of head with shallow anterior median furrow. Lateral marginal ridge discontinuous. Posterior margin continuous, convex (Fig. 1A).

On each side of head, 1 + 3, 3, 1 oval to rounded ocelli arranged in three irregular rows; posterior ocellus large; ocelli adjacent to the Tömösváry organ slightly smaller. Seriate ocelli domed, translucent, usually darkly pigmented. Tömösváry organ at anterolateral margin of the cephalic plate, larger than the adjacent ocelli.

***Coxosternite subtrapezoidal*** (Fig. 1C) with narrow dental straight dental margin, anterior margin narrow, lateral margins of the coxosternite longer than medial margins. Median diastema shallow; anterior margin with 2 + 2 small blunt teeth that are encircled by a narrow rim. Porodont thick and strong, just posterolateral and ventral from the lateral tooth, bulged at base (Fig. 1C). Scattered short and long setae on the ventral side of coxosternite, longer setae near the porodont.

All tergites with numerous minute setae scattered on surface, several setae on anterior and posterior angles of each tergite and lateral borders, dorsum slightly convex; T1 slightly narrower posterolaterally than anterolaterally, generally trapezoidal, slightly narrower than the cephalic plate and T3, cephalic plate slightly about the same size as T3. Lateral marginal ridges of all tergites continuous. Posterior marginal ridges of TT 1, 3 and 5 concave, continuous, posterior marginal ridges of TT 7, 8, 10, 12 and 14 slightly concave, discontinuous. Posterior angles of all tergites rounded, without triangular projections.

Sternites smooth, posterior part of sternites narrower than anterior, generally trapezoidal. Sternites with 8 short to long setae on anterior corners and anterior lateral borders, 2 setae on posterior lateral borders.

***Legs*** slender, tarsal articulation well defined on legs 1–15. All legs with fairly long curved claws; pretarsus of legs 1–13 with a slightly curved, long, principal claw and anterior and posterior accessory spines, anterior accessory spines slightly longer and slender, ca 0.56 the length of principal claw, posterior one stouter, ca 0.43 the length of principal claw, forming slightly larger angles with tarsal claws; leg 14 with only posterior spines; leg 15 lacking accessory spines. Dense glandular pore on surface of femur, tibia and tarsi of legs 14 and 15. Long setae sparsely scattered over surface of prefemur, femur, tibia, and tarsi of legs 14–15, more setae on the tarsal surface. 6–7 thicker setae arranged in one row on the ventral surface of tarsus 1 of legs 1–13, 6–7 pairs of thicker setae arranged in two rows on the ventral surface of tarsus 2 of legs 1–13. Legs 14 and 15 thicker and stronger than the anterior pairs. 15^th^ leg 40.6% of body length, tarsus 1 3.3 times longer than wide, tarsus 2 44.4% length of tarsus on leg 15. No modification on legs 14 and 15 in males. Leg plectrotaxy as in Table 1.

**Table 1.** Lithobius (Ezembius) hualongensis sp. nov.: plectrotaxy of legs. Letters in brackets indicate spines on one leg of pair, or in one specimen.

**Legs**	**Ventral**					**Dorsal**				
	**C**	**Tr**	**P**	**F**	**Ti**	**C**	**Tr**	**P**	**F**	**Ti**
1	–	–	mp	amp	am	–	–	mp	ap	a
2–4	–	–	(a)mp	amp	am	–	–	(a)mp	ap	ap
5	–	–	amp	amp	am	–	–	(a)mp	ap	ap
6–11	–	–	amp	amp	am	–	–	amp	ap	ap
12	–	m	amp	amp	am	a	–	amp	ap	ap
13	–	m	amp	amp	am	a	–	amp	ap	ap
14	–	m	amp	amp	am	a	–	amp	p	p
15	–	m	amp	amp	a	a	–	amp	p	–

Coxal pores 4655 round or slightly oval, variable in size, arranged in a row. Coxal pore field set in a relatively shallow groove, the coxal pore-field fringe with prominence. Prominence with short to moderately long setae sparsely scattered over the surface.

***Male posterior segment*.** Male S15 subtrapeziform, posterior margin narrower than anterior, sparsely covered with short to long setae on ventral side of S15 and lateral and posterior borders (Fig. 1D); sternite of genital segment obviously smaller than the female, sclerotized; posterior margin deeply concave between the gonopods, without medial bulge. Long setae scattered on the ventral surface of the genital segment. Gonopods short, appearing as a small hemispherical bulge, with one long setae, apically slightly sclerotized (Fig. 1D).

***Female posterior segment*.** Female S15 anterior margin broader than posterior, generally trapezoidal, posteromedially straight, S15 with short to long setae on the ventral surface and lateral and posterior borders. Posterior margin of genital sternite deeply concave between condyles of gonopods, except for a small, median rhomboid bulge. Short to long setae sparsely scattered on ventral surface of genital segment. Gonopods: first article fairly broad, bearing 17–20 short to moderately long setae, arranged in four irregular rows; with 3 + 3, moderately long and slender spurs, inner spur smaller than the outer (Fig. 1E); second article with 6–7 long setae, arranged in two irregular rows, with 10 short to long dorsal lateral setae, stouter than the general setae (Fig. 1F); third article with 6 long setae arranged in one irregular row, and 7 short setae on dorsal lateral side (Fig. 1F); third article terminal claw simple and sharp, having a very small triangular protuberance on ventral side (Fig. 1E).

###### Variations.

Body length 12.31–16.15 mm; ocelli 1 + 3, 3, 1 or 1 + 4, 3, 3; coxal pores 6666 and 6777 in female and 4654 and 4655 in male.

###### Remarks.

Morphologically, the new species can be easily distinguished from the seven species in the subgenus from Qinghai Province, L. (E.) asulcutus, L. (E.) rarihirsutipes, L. (E.) femorisulcutus, L. (E.) longibasitarsus, L. (E.) datongensis, L. (E.) maqinensis and L. (E.) dulanensis, by the 3 + 3 coniform spurs on female gonopods contrary to 2 + 2 coniform spurs (Table 2).

**Table 2.** Main morphological characters of Chinese species of subgenus Lithobius (Ezembius) Chamberlin, 1919 from Qinghai Province.

Characters	* asulcutus *	* rarihirsutipes *	* femorisulcutus *	* longibasitarsus *	* datongensis *	* maqinensis *	* dulanensis *	*hualongensis* sp. nov.	*sui* sp. nov.
Sources	Zhang 1996	Zhang 1996	Zhang 1996	Qiao et al. 2018b	Qiao et al. 2018b	Qiao et al. 2019a	Qiao et al. 2019b	this paper	this paper
Body length (mm)	13–15	11–12	15–18	17–18	12.3–14.2	13.1–14.6	about 20.5	12.31–16.15	12.15–18.85
Number of antennal articles	20 + 20	20 + 20	20 + 20	20 + 20	20 + 20	20 + 20	20–21	20 + 20	20 + 20
Number, arrangement of ocelli	10, in 3 rows	11, in 3 rows	10–14, in 3 rows	10–14, in 3 rows	9–10 ocelli, in 3 broken rows	10–12, in 3 rows	11, in 3 rows	8–11, in 3 rows	9–10, in 3 rows
Posterior ocellus	round, comparatively large	oval to round, large	comparatively large	posterior ocellus is biggest	slightly larger than posterosuperior ocellus	posterior ocellus is biggest	posterior ocellus and posterosuperior ocellus comparatively large	terminal two ocelli comparatively large	terminal two ocelli comparatively large
Seriate ocelli	ones near ventral margin moderately small	ones near ventral margin moderately small	ones near ventral margin moderately small	ones near ventral margin moderately small	ones near ventral margin moderately small	ones near ventral margin moderately small	second row smaller than first, third smallest	ones near ventral margin moderately small	ones near ventral margin moderately small
Tömösváry’s organ	round, smaller than adjoining ocelli	rounded, slightly, smaller than adjoining ocelli	slightly larger than adjoining ocelli	slightly smaller than adjoining ocelli	larger than adjoining ocelli	almost same size as adjacent ocelli	oval and slightly smaller than adjoining ocelli	obviously larger than adjoining ocelli	obviously larger than adjoining ocelli
Number and shape of coxosternal teeth	2 + 2 subtriangular teeth	2 + 2, subtriangular teeth	2 + 2	2 + 2 or 3 + 2	2 + 2	2 + 2, small coniform teeth	2 + 2, coniform, moderately robust teeth	2 + 2, small coniform teeth	3 + 3, inner tooth smaller than outer tooth
Porodont	long and slender, lying posterolateral to lateral-most teeth	long and slender, lying posterolateral to lateral-most tooth	long and strong, lying posterolateral to lateral-most tooth	thick and strong separated from lateral tooth ventrolaterally	setiform, separated from lateral tooth laterally	setiform, lying posterolateral to lateral-most tooth	long and strong, lying posterolateral to lateral-most tooth	thick and long, lying posterolateral to lateral-most tooth	thick and long, lying posterolateral to lateral-most tooth
Tergites	smooth	smooth	smooth	smooth	smooth	smooth	smooth	with numerous minute setae scattered on surface	with numerous minute setae scattered on surface
Number of coxal pores	4–7, 4544, 4554, 5665, 5766	not reported	5544, 5554, 5555, 5564	4–6	4–7	4–6	5–7, 5667, 5666	6666 and 6777 in female and 4654 and 4655 in male	5664, 5665, 7775 and 8875 in female and 6886 and 7665 in male.
Shape of coxal pores	not reported	not reported	round	circular	round	round	circular to ovate	round or slightly ovate	round or slightly ovate
Tarsus 1–tarsus 2 articulation on legs 1–13	not well defined	well defined	well defined	well defined	well defined	not well defined	not well defined	well defined	well defined
Male 14^th^ leg	slightly thicker and stronger than other legs	markedly thicker and stronger than 1–13 legs	slightly thick and strong than 1–13 legs	moderately thick and strong than 1–13 legs	moderately thick and strong than 1–13 legs	moderately thick and strong than 1–13 legs	slightly thick and strong than 1–13 legs	slightly thick and strong than 1–13 legs	slightly thick and strong than 1–13 legs
Male 15^th^ leg	slightly thicker and stronger than other legs	markedly thicker and stronger than 1–13 legs	slightly thick and strong than 1–13 legs	moderately thick and strong than 1–13 legs	moderately thick and strong than 1–13 legs	moderately thick and strong than 1–13 legs	slightly thick and strong than 1–13 legs	slightly thick and strong than 1–13 legs	slightly thick and strong than 1–13 legs
Dorsal sulci on male 15^th^ legs	absent	two distinct, dorsal sulci on femur	distinct, dorsal sulcus on tibia and tarsus 1	absent	absent	absent	absent	absent	absent
DaC spine	on 12^th^–15^th^ legs	on 12^th^–15^th^ legs	on 11^th^–15^th^ legs	on 12^th^–15^th^ legs	on 12^th^–15^th^ legs	on 11^th^–15^th^ legs	on 11^th^–15^th^ legs	on 12^th^–15^th^ legs	on 12^th^–15^th^ legs
14^th^ accessory spur	present	present	present	present	present	anterior accessory spur absent, posterior accessory spur present	anterior accessory spur absent, posterior accessory spur present	anterior accessory spur absent, posterior accessory spur present	anterior accessory spur absent, posterior accessory spur present
15^th^ accessory spur	absent	absent	present	present	present	absent	absent	absent	anterior accessory spur absent, posterior accessory spur present
Number and shape of spurs on female gonopods	2 + 2 coniform spurs	2 + 2 conical spurs	2 + 2 conical spurs	2 + 2 conical spurs	2 + 2 conical spurs	2 + 2 conical spurs	2 + 2 conical spurs	3 + 3 coniform spurs, inner spur moderately smaller than outer one	3 + 3 coniform spurs, inner spur moderately smaller than outer one
Dorsal side of second article of female gonopods	not reported	not reported	not reported	four short setae and three long setae on dorsolateral ridge	five long curved spines on dorsolateral side	not reported	with six dorsolateral setae	with 10 short to long dorsal lateral setae, stouter than general setae	with 10 short dorsal lateral setae, stouter than general setae
Apical claw of female gonopods (and lateral denticles)	simple	simple, having small sharp teeth on the inner side	simple	simple, having small triangular protuberance on ventral side	simple, bearing very small triangular protuberance on ventral side	simple, having very small triangular protuberance on ventral side	simple	simple, having very small triangular protuberance on ventral side	simple, having very small triangular protuberance on ventral side
Male gonopods	short and small bulge, with two long setae	with small bulge, with 3 long setae	not reported	single small semicircular article with 3–5 setae on its surface	hemispherical, with three setae	small, oblique apically, with 2 setae	small, one-segmented, with two long setae	short and small bulge, having long seta, apically slightly sclerotized	short and small, with two long setae on terminal segment

###### Etymology.

The new species is named from the type locality.

###### Habitat.

The four specimens here examined (3 ♂♂, 1 ♀) were collected under granular gravel on the alpine meadows composed mainly of Poaceae, Cyperaceae, Fabaceae, Polygonaceae, Chenopodiaceae, Asteraceae, Rosaceae, Liliaceae and Cucurbitaceae. The region is located on the upper reaches of the Yellow River Valley and features an arid climate, with mean annual precipitation 451.2 mm and average annual temperature 2.8 °C (http://data.cma.cn/data/weatherBk.html).

##### Lithobius (Ezembius) sui sp. nov.

Taxon classificationAnimaliaLithobiomorphaLithobiidae

26FDF703-254B-520D-8955-44284E3C385E

http://zoobank.org/71D02371-BD0E-43CA-8986-5775CC49405C

Fig. 2A–G ; Tables 2 , 3

###### Type materials.

***Holotype***: ♀ (MHA8), Minhe County, Qinghai Province, 36.12076N, 102.7809E, 2280 m, a.s.l., 24 October 2010, leg. Lin Gong-Hua. ***Paratypes***: 1 ♂ (MHA6), 2♀♀ (MHA1, MHA5), all from the same locality.

###### Diagnosis.

A Lithobius (Ezembius) species with body length 12.15–18.85 mm, antennae of 20 + 20 articles; 9–10 ocelli on each side, arranged in 3 irregular rows, terminal two ocelli comparatively large; Tömösváry’s organ distinctly larger than the adjoining ocelli; 3 + 3 coxosternal teeth; porodonts posterolateral and ventral to the lateral-most tooth; posterior angles of all tergites without triangular projections; 4–8 coxal pores oval to round, arranged in one row; female gonopods with 3 + 3 moderately large, coniform spurs; terminal claw of the third article simple, with a very small triangular protuberance on basal ventral side; male gonopods short and small, with two long setae on the terminal segment.

###### Description.

**Female** (Fig. 2A). Body length 12.15 mm, cephalic plate 1.54 mm long, 1.69 mm broad.

**Figuree 2.**
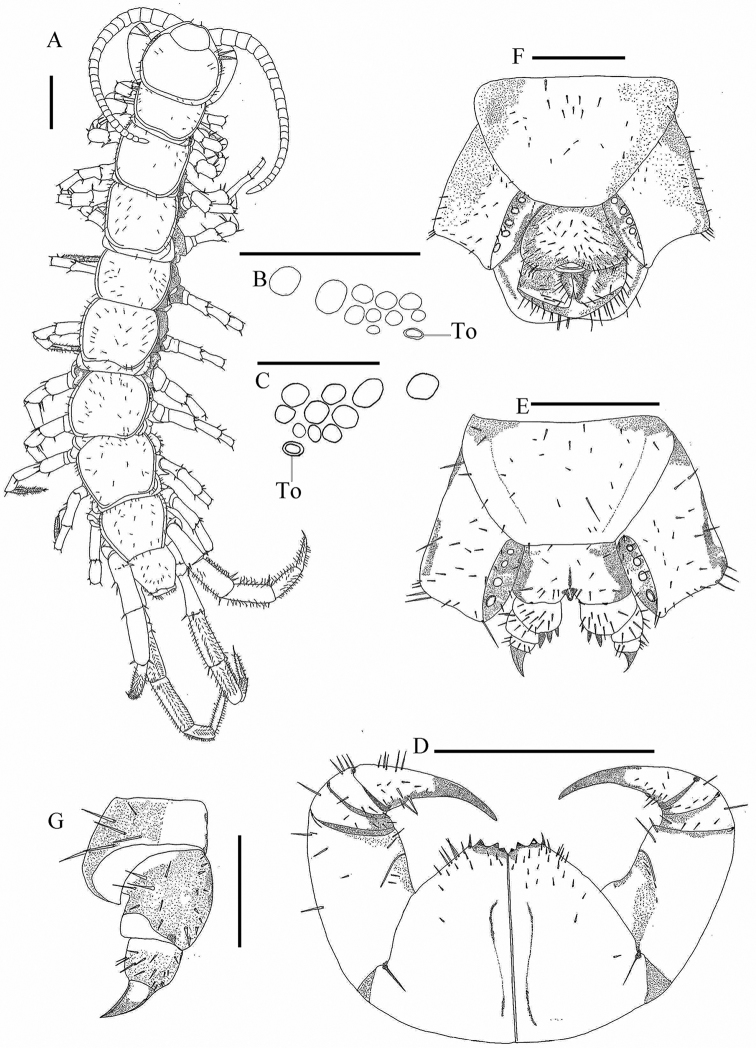
Lithobius (Ezembius) sui sp. nov. **A, B, D, E, G** holotype, female: **A** habitus, dorsal view **B** ocelli and Tömösváry’s organ (**To**), lateral view **D** forcipular coxosternite, ventral view **E** female posterior segments and gonopods, ventral view **G** female gonopods, dorsal lateral view **C, F** paratype, male: **C** ocelli and Tömösváry’s organ (**To**), lateral view **F** posterior segments and gonopods, ventral view.Scale bars: 1 mm (**A, D**); 500 μm (**E, F**); 250 μm (**B, C, G**).

***Coloration*.** Body yellow-brown, cephalic plate and antennae light yellow-brown with reddish hue. Pleural region pale grey. Sternites yellow-brown. Distal part of forcipules red-brown, with basal and proximal parts of forcipules and forcipular coxosternite yellow-brown. Legs 1–15 light yellow-brown.

***Antennae*** composed of 20 + 20 articles extending back to posterior part of T3, basal article about the same width as length, second article slightly longer than wide, third article slightly wider than long, with following articles tapering, distal-most article 2.9 times as long as wide; abundant setae on antennal surface, gradual increase in density of setae basally to distally to about fourth articles.

***Cephalic plate*** smooth, convex. Frontal marginal ridge of head with shallow anterior median furrow. Lateral marginal ridge discontinuous. Posterior margin continuous, slightly convex (Fig. 2A).

On each side of head, 1 + 4, 4, 1 oval to rounded ocelli (Fig. 2B), arranged in three irregular rows; posterior ocellus large; ocelli adjacent to the Tömösváry organ slightly small. Seriate ocelli domed, translucent, usually darkly pigmented. Tömösváry organ at anterolateral margin of the cephalic plate, larger than the adjacent ocelli (Fig. 2B-To).

***Coxosternite subtrapezoidal*** (Fig. 2D), anterior margin narrow, lateral margins of the coxosternite longer than medial margins. Median diastema moderately shallow, V-shaped; anterior margin with 3 + 3 subtriangular blunt teeth, inner tooth smaller. Porodont thick and strong, just posterolateral and ventral from the lateral tooth, bulged at base (Fig. 2D). Scattered short setae on the ventral side of coxosternite, longer setae near the porodont.

All tergites with numerous minute setae scattered on surface, several setae on anterior and posterior angles of each tergite and lateral borders, dorsum slightly convex; T1 narrower posterolaterally than anterolaterally, generally trapezoidal, narrower than the cephalic plate and T3, cephalic plate slightly wider than T3. Lateral marginal ridges of all tergites continuous. Posterior marginal ridges of TT 1 and 3 slightly concave, continuous, posterior marginal ridges of TT 5, 7, 8, 10, 12 and 14 concave, discontinuous. Posterior angles of all tergites rounded, without triangular projections.

Sternites smooth, posterior part of sternites narrower than anterior, generally trapezoidal. Sternites with 2–7 short to long setae on anterior corners and anterior lateral borders, the same with posterior lateral and posterior angles.

***Legs*** slender, tarsal articulation well defined on legs 1–15. All legs with fairly long curved claws; pretarsus of legs 1–13 with a slightly curved, long, principal claw and anterior and posterior accessory spines, anterior accessory spines slightly longer and slender, ca 0.50 the length of principal claw, posterior one stouter, ca 0.33 the length of principal claw, forming slightly larger angles with tarsal claws; legs 14 and 15 with only posterior spines. Dense glandular pore on surface of femur, tibia and tarsi of legs 14 and 15. Long setae sparsely scattered over surface of prefemur, femur, tibia, and tarsi of legs 14–15, more setae on the tarsal surface. 7–9 thicker setae arranged in one row on the ventral surface of tarsus 1 of legs 1–13, 7–8 pairs of thicker setae arranged in two rows on the ventral surface of tarsus 2 of legs 1–13. Legs 14 and 15 thicker and stronger than the anterior pairs. 15^th^ leg 39.24% of body length, tarsus 1 3.8 times longer than wide, tarsus 2 44.4% length of tarsus on leg 15. Leg plectrotaxy as in Table 3.

**Table 3.** Lithobius (Ezembius) sui sp. nov.: plectrotaxy of legs. Letters in brackets indicate spines on one leg of pair, or in one specimen.

**Legs**	**Ventral**					**Dorsal**				
	**C**	**Tr**	**P**	**F**	**Ti**	**C**	**Tr**	**P**	**F**	**Ti**
1	–	–	mp	amp	am	–	–	(a)mp	ap	a
2–4	–	–	mp	amp	am	–	–	(a)mp	ap	ap
5–7	–	–	mp	amp	am	–	–	amp	ap	ap
8–11	–	–	amp	amp	am	–	–	amp	ap	ap
12	–	m	amp	amp	am	a	–	amp	ap	ap
13	–	m	amp	amp	am	a	–	amp	(a)p	ap
14	–	m	amp	amp	am	a	–	amp	p	p
15	–	m	amp	amp	a	a	–	amp	p	–

Coxal pores 4–8 round or slightly oval, variable in size, arranged in a row. Coxal pore field set in a relatively shallow groove, the coxal pore-field fringe with prominence. Prominence with short to moderately long setae sparsely scattered over the surface.

***Female posterior segment*.** Female S15 anterior margin broader than posterior, generally trapezoidal, posteromedially straight, S15 with short to long setae on the ventral surface and lateral and posterior borders. Posterior margin of genital sternite deeply concave between condyles of gonopods, except for a small, median rhomboid bulge. Short to long setae sparsely scattered on ventral surface of genital segment. Gonopods: first article fairly broad, bearing 9–11 short to moderately long setae, arranged in three irregular rows; with 3 + 3, moderately long and slender, bullet-shape spurs, inner spur smaller than the outer (Fig. 2E); second article with 6–7 long setae, arranged in two irregular rows, with 10 short dorsal lateral setae, stouter than the general setae (Fig. 2G); third article with 6 long setae arranged in one irregular row, and 7 short setae on dorsal lateral side (Fig. 2G); third article terminal claw simple and sharp, having a very small triangular protuberance on ventral side (Fig. 2E).

***Male posterior segment*.** Male S15 subtrapeziform, posterior margin narrower than anterior, sparsely covered with short to long setae on ventral side of S15 and lateral and posterior borders (Fig. 2F); sternite of genital segment sclerotized; posterior margin deeply concave between the gonopods, without medial bulge. Long setae scattered on the ventral surface of the genital segment. Gonopods short, appearing as a small hemispherical bulge, with two long setae, apically slightly sclerotized (Fig. 2F). No modification on legs 14 and 15 in males.

###### Variations.

Body length 12.15–18.85 mm; ocelli 1 + 4, 4, 1, 1 + 4, 3, 2 or 1 + 4, 3, 1; coxal pores 5664, 5665, 7775 or 8875 in female and 6886, 7665 in male.

###### Remarks.

The two new species are very similar in morphology, especially in both having numerous minute setae scattered on surface of all tergites, but can be distinguished by the number of coxosternal teeth: L. (E.) hualongensis sp. nov. has 2 + 2 while L. (E.) sui sp. nov. has 3 + 3.

###### Etymology.

The specific name is a patronym in honor of the zoologist Dr Jianping Su, Academician at the Chinese Academy of Sciences.

###### Habitat.

The four specimens here examined (1 ♂, 3 ♀♀) were collected under granular gravel on the alpine meadows composed mainly of Gramineae, Cyperaceae, Leguminosae, Polygonaceae, Chenopodiaceae, Compositae, Rosaceae, Liliaceae and Cucurbitaceae. This region adjacent to Hualong County in the west features plateau continental, with mean annual precipitation 338.1 mm and average annual temperature 8.3 °C (http://data.cma.cn/data/weatherBk.html).

#### Key to the Chinese species of Lithobius (Ezembius)

**Table:** 

1	Posterior angles of tergites with triangular projections	***L.* (*E.*) *sulcipes* Attems, 1927**
–	Posterior angles of tergites rounded, without projections	**2**
2	At most four ocelli on each side of cephalic plate	***L.* (*E.*) *parvicornis* (Porat, 1893)**
–	At least five ocelli on each side of cephalic plate	**3**
3	Cephalic plate with scattered, rough puncta and tergite with distinct puncta	***L.* (*E.*) *rhysus* Attems, 1934**
–	Cephalic plate and tergite without any puncta	**4**
4	All ocelli subequal in size	***L.* (*E.*) *sulcifemoralis* Takakuwa & Takashima, 1949**
–	All ocelli not subequal in size	**5**
5	Terminal two ocelli comparatively large	**6**
–	Terminal one ocellus comparatively large	**13**
6	Ocelli arranged in two rows	***L.* (*E.*) *laevidentata* Pei, Ma, Hou, Zhu & Gai, 2015**
–	Ocelli arranged in three rows	**7**
7	3 + 3 coxosternal teeth	**8**
–	2 + 2 coxosternal teeth	**9**
8	First article of female gonopods with 3 + 3 spurs	**L. (E.) sui sp. nov.**
–	First article of female gonopods with 2 + 2 spurs	***L.* (*E.*) *multispinipes* Pei, Lu, Liu, Hou, Ma & Zapparoli, 2016**
9	Tömösváry’s organ larger than adjoining ocellus	**10**
–	Tömösváry’s organ smaller than adjoining ocellus	**11**
10	First article of female gonopods with 3 + 3 spurs	**L. (E.) hualongensis sp. nov.**
–	First article of female gonopods with 2 + 2 spurs	***L.* (*E.*) *bilineatus* Pei, Ma, Zhu & Gai, 2014**
11	Apical claw of female gonopods simple, without inner small subtriangular teeth	**L. (E.) tetraspinus Pei, Lu, Liu, Hou & Ma, 2018**
–	Apical claw of female gonopods simple, with inner small subtriangular teeth	**12**
12	Number of antennal articles 23 + 23	***L.* (*E.*) *anabilineatus* Ma, Pei, Hou, Zhu & Gai, 2015**
–	Number of antennal articles 20 + 20–21 + 21	***L.* (*E.*) *dulensis* Qiao, Ma, Pei, Zhang & Su, 2019**
13	Only five ocelli on each side of cephalic plate	***L.* (*E.*) *chekianus* Chamberlin & Wang, 1952**
–	At least six ocelli on each side of cephalic plate	**14**
14	Tömösváry’s organ smaller than adjoining ocellus.	**15**
–	Tömösváry’s organ larger or subequal in size as adjoining ocellus	**21**
15	First article of female gonopods with 3 + 3 or 4 + 4 spurs	**16**
–	First article of female gonopods with 2 + 2 spurs	**17**
16	Apical claw of female gonopods simple, with innersmall subtriangular teeth	***L.* (*E.*) *bidens* Takakuwa, 1939**
–	Apical claw of female gonopods simple, without inner small subtriangular teeth	***L.* (*E.*) *insolitus* Eason, 1993**
17	Terminal claw of female gonopods bipartite	***L.* (*E.*) *anasulcifemoralis* Ma, Pei, Wu & Gai, 2013**
–	Terminal claw of female gonopods simple	**18**
18	Terminal claw of female gonopods simple, with inner small triangular teeth	**19**
–	Terminal claw of female gonopods simple, without inner small triangular teeth	**20**
19	Body length 11–12 mm, 15^th^ accessory spur absent	**L. (E.) rarihirsutipes Zhang, 1996**
–	Body length 17–18 mm, 15^th^ accessory spur present	**L. (E.) longibasitarsus Qiao, Qin, Ma, Zhang, Su & Lin, 2018**
20	DaC spine on 12^th^ –15^th^ legs	**L. (E.) asulcutus Zhang, 1996**
–	DaC spine absent	***L.* (*E.*) *giganteus* Sseliwanoff, 1881**
21	Six ocelli on each side of cephalic plate	***L.* (*E.*) *gantoensis* Takakuwa & Takashima, 1949**
–	At least seven ocelli on each side of cephalic plate	**22**
22	Ocelli arranged in two rows	**L. (E.) irregularis Takakuwa & Takashima, 1949**
–	Ocelli arranged in three rows	**23**
23	First article of female gonopods with 3 + 3 spurs	**24**
–	First article of female gonopods with 2 + 2 spurs	**25**
24	DaC spine on 14^th^–15^th^ legs	***L.* (*E.*) *lineatus* Takakuwa, 1939**
–	DaC spine on 12^th^–15^th^ legs	***L.* (*E.*) *mandschreiensis* Takakuwa, 1940**
25	Terminal claw of female gonopods tridentate	***L.* (*E.*) *zhui* Pei, Ma, Shi, Wu & Gai, 2011**
–	Terminal claw of female gonopods simple	**26**
26	Terminal claw of female gonopods simple, without inner small triangular teeth	***L.* (*E.*) *femorisulcutus* Zhang, 1996**
–	Terminal claw of female gonopods simple, with inner small triangular teeth	**27**
27	Tarsal articulation on legs 1–13 well defined, 14^th^ accessory spur present	***L.* (*E.*) *datongensis* Qiao, Qin, Ma, Zhang, Su & Lin, 2018**
–	Tarsal articulation on legs 1–13 not well defined, only 14^th^ posterior accessory spur present	***L.* (*E.*) *maqinensis* Qiao, Qin, Ma & Zhang, 2019**

## Funding

This work was financially supported by the the Strategic Priority Research Program of the Chinese Academy of Sciences [XDA23060602, XDA2002030302]; National Key R&D Program of China [2017YFC0506405]; and Qinghai Key R&D and Transformation Program [2019-SF-150].

## Supplementary Material

XML Treatment for Lithobius (Ezembius) hualongensis

XML Treatment for Lithobius (Ezembius) sui
